# Mosquitofish (*Gambusia affinis*) Preference and Behavioral Response to Animated Images of Conspecifics Altered in Their Color, Aspect Ratio, and Swimming Depth

**DOI:** 10.1371/journal.pone.0054315

**Published:** 2013-01-16

**Authors:** Giovanni Polverino, Jian Cong Liao, Maurizio Porfiri

**Affiliations:** Department of Mechanical and Aerospace Engineering, Polytechnic Institute of New York University, Brooklyn, New York, United States of America; Tulane University Medical School, United States of America

## Abstract

Mosquitofish (*Gambusia affinis*) is an example of a freshwater fish species whose remarkable diffusion outside its native range has led to it being placed on the list of the world’s hundred worst invasive alien species (International Union for Conservation of Nature). Here, we investigate mosquitofish shoaling tendency using a dichotomous choice test in which computer-animated images of their conspecifics are altered in color, aspect ratio, and swimming level in the water column. Pairs of virtual stimuli are systematically presented to focal subjects to evaluate their attractiveness and the effect on fish behavior. Mosquitofish respond differentially to some of these stimuli showing preference for conspecifics with enhanced yellow pigmentation while exhibiting highly varying locomotory patterns. Our results suggest that computer-animated images can be used to understand the factors that regulate the social dynamics of shoals of *Gambusia affinis.* Such knowledge may inform the design of control plans and open new avenues in conservation and protection of endangered animal species.

## Introduction

In the last few decades, conservationists have increasingly called for action to protect native animal species and habitats from “biological invasions” of invasive alien species. The presence of animals and plants that adversely affect both the ecological integrity and the local economy in areas in which they are not indigenous is common across the globe [1],[2],[3],[4],[5],[6]. Their dispersal is often due to voluntary and involuntary human activities [6],[7]. The negative impact of invasive alien species on biodiversity is second only to habitat loss [8],[9]. In the United States, the economic and environmental costs due to these species were recently estimated to be $120 billion per year [10] and the extinction of 750,000 species [9]. At least 138 of the invasive alien species in the United States are fish [11],[12] (these statistics have presumably increased in the two decades since their last assessment).

*Gambusia affinis* is an example of a social freshwater fish native to the eastern United States whose diffusion was actively driven by humans in the nineteenth century for its use as mosquito control agent in wetland areas [2],[13],[14],[15],[16]. Such use has resulted in the common terminology “mosquitofish”. The remarkable invasion of mosquitofish in the environment and their negative impact on indigenous animal communities [7],[13],[15],[16],[17],^18^,[19] has led to it being placed on the International Union for Conservation of Nature’s list of the world’s hundred worst invasive alien species [6]. Specifically, mosquitofish are responsible for the impairment of foraging success, decreased survival rate, and reduction in reproduction rate of several native fish of comparable size [13],[18],[20],[21],[22],[23] and amphibians’ tadpoles [17],[24], which cannot effectively compete with this highly adaptive colonizer.

Mosquitofish exhibit both social and anti-social behavior between genders [15],[25],[26] and their interactions with other fish of similar size are generally competitive if not predatorial [18],[21],[22],[27]. The investigation of the behavioral response of mosquitofish in controlled environments can aid a better understanding of the determinants of their social interactions in ecological contexts. For example, social recognition in mosquitofish has been found to be primarily affected by visual [28],[29] and chemical cues [29], whose synthesis allows individuals to respond quickly to the presence of conspecifics and predators [29]. Increasing the group size has been observed to improve the speed and the accuracy of predator detection [30]. Furthermore, it has recently been demonstrated that mosquitofish shoaling tendency is determined by interactions between nearest neighbors in the form of attraction forces and repulsion mediated by changes in speed [31]. Males’ social rank and females’ mate choice have been demonstrated to be correlated with color patterns [28],[32]. Individual personality traits in mosquitofish have been shown to be persistent in time and correlated to the social or aggressive tendencies of the individuals [33].

Computer-animated images have recently emerged as a powerful tool to investigate fish behavior by administering controlled stimuli [34],[35],[36],[37],[38],[39]. The use of computer animations allows for the isolation of specific visual cues from potential confounds arising from auditory, electrical, flow, and chemical confounds while offering a precise, consistent, inexpensive, high-throughput, and easily manageable non-invasive methodology to investigate fish behavior [39], especially when coupled with automated video tracking [40],[41]. The use of such stimuli has been successfully implemented to investigate the behavioral response of several social species, such as three-spined sticklebacks [42], swordtails [43],[44], pipefish [45], cichlids [46], tiger barb [47], and zebrafish [48],[49],[50],[51],[52],[53].

This study seeks to evaluate how systematic changes of the visual characteristics of animated images of mosquitofish shoals versus unaltered images of mosquitofish shoals may affect isolated focal fish. Specifically, we investigate mosquitofish preference and locomotory patterns in a dichotomous choice test, in which images are systematically altered in their color, aspect ratio, and swimming depth in the water column.

## Materials and Methods

The experiment described in this work was approved by the Polytechnic Institute of New York University (NYU-Poly) Animal Welfare Oversight Committee AWOC-2012-102. Both the housing and the experimental procedure were designed to minimize stress in the animals.

### Animals and Housing

One hundred mosquitofish (*Gambusia affinis*) were procured from an online aquarium source (LiveAquaria.com, Rhinelander, Wisconsin, USA). Fish were acclimated for a minimum of two weeks in the vivarium facility housed in the Department of Mechanical and Aerospace Engineering at NYU-Poly prior to the experiments. According to their mean body length, which was circa 3±0.5 cm, mosquitofish were sexually mature young adults [2]. Individuals of this size are known to display prominent shoaling tendencies [15],[25]. Following [31], sixty females of mosquitofish were selected for this study based on their strong gregarious tendency [25],[26],[54]. Mosquitofish were housed in a holding tank 90 cm long, 30 cm wide, and 40 cm high, with a capacity of 110 l and a fish density equal to 0.54 fish/l. The water temperature was maintained at 27±0.5°C and pH at 7.2. The holding tank was equipped with an external overflow filtration system (Marineland, Emperor 400 BIO-Wheel) and was illuminated by full spectrum fluorescent lights for ten hours each day in accordance with the circadian rhythm of the species (see [15],[55]). Fish were fed with commercial flake food (Hagen Corp., Nutrafin max, USA) after the end of daily experimentation.

### Apparatus

**Figure 1:**
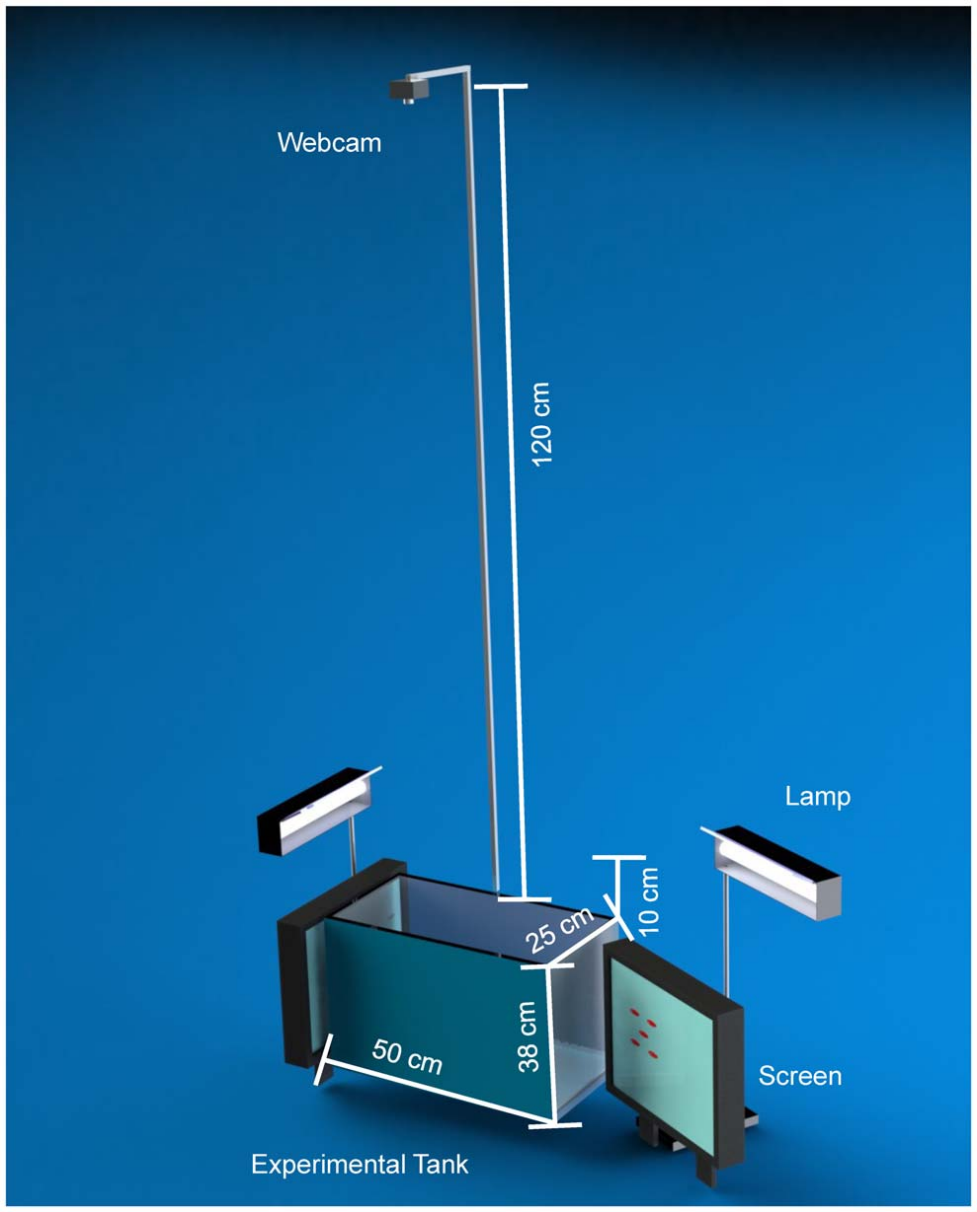
Schematic of the experimental setup. The test tank was equipped with two monitors, two lamps, and a bird’s-eye camera (one monitor and a lamp are pulled aside in this picture to show the projected animated images). The blue background of the images was compatible with the tank background. The bird’s-eye camera recorded the fish motion from above while the two lamps provided homogeneous illumination.

The test tank used for the experiments was 50 cm long, 25 cm wide, and 30 cm high, with a capacity of 36 l. Water quality and temperature of the holding and test tanks were kept the same by using comparable external overflow filter and heater (Elite, A750). Both the filter and the heater were removed from the test tank during the trials to facilitate the localization of fish. The test tank was illuminated by two 8 W fluorescent lamps (All-Glass Aquarium, preheat aquarium lamp, U.K.) placed over the two short sides of the test tank. A bird’s-eye view of the test tank was obtained by positioning a webcam (Logitech, Webcam Pro 9000) approximately 120 cm above the water’s surface to minimize the distortion produced by the curvature of the lens while providing an ample resolution for fish tracking. The test tank was equipped with two monitors (Dell E117FPc LCD Monitor, Round Rock, TX, USA) positioned adjacent to the two short sides. The bottom surface of the tank was covered with an opaque plastic blue sheet to optimize fish detection based on the comparison with preliminary experiments using either white or black colored surfaces. To isolate the experimental environment and provide a homogeneous background to the fish, the two longitudinal sides of the test tank were also covered with the same opaque plastic sheet and the background color of the screens was selected to be blue. Figure 1 shows a schematic of the experimental setup.

### Animated Images

**Figure 2:**
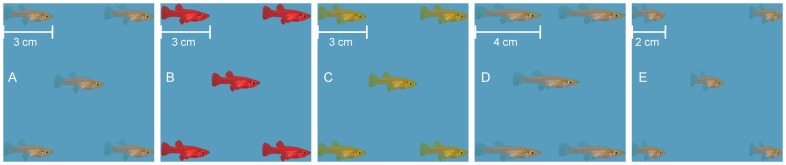
Array of animated images used in the experiments. Naturally colored mosquitofish (A) were juxtaposed to red (B) and yellow (C) colored images, respectively. The effect of the image aspect ratio was investigated by confronting images of mosquitofish with natural body shape (A) with longitudinally elongated (D) and compressed (E) images. Dimensions of the images are indicated in the figure.

Five replicas of a picture of an adult female mosquitofish of three cm body length, viewed in the sagittal plane, were oriented in a face-centered cubic system (two-dimensional) to form a virtual shoal whose inter-fish distances were maintained constant in each experimental condition following the experimental design of [48] for zebrafish (see Figure 2A). The group size is compatible with other studies on mosquitofish shoaling and perceptual numerosity [56]. Such unaltered virtual shoal was modified by either varying the aspect ratio or the color of the mosquitofish. Compressed or elongated mosquitofish were obtained by changing the body length to two or four cm (see Figure 2E and Figure 2D). Color variations consisted of changing the pigmentation of mosquitofish in the unaltered images into yellow or red (see Figure 2C and Figure 2B). Between conditions, the geometric extent of the virtual shoal was kept constant at nine cm × nine cm and inter-fish distance was consistently varied to accommodate for alterations of mosquitofish aspect ratio. Specifically, the average inter-fish distance was three cm for the natural aspect ratio and four or two cm for elongated or compressed images, respectively (see Figure 2).

For each screen, the virtual shoal horizontally traversed a 25 cm×26 cm focal region in multiple sweeps corresponding to the portion of tank side wall below the water surface. The starting location of the virtual shoal image was randomized in all videos. The speed of the virtual shoal was kept constant in each sweep across the screen and was varied between sweeps in each video. Specifically, the speed was maintained at a mean value of 1.5 cm/s and a standard deviation of 1 cm/s in each video. During each sweep, the center of the virtual shoal was either 6 cm (shoal moving in the tank top half) or 18 cm (shoal moving in the tank bottom half) from the water surface. In most videos, the vertical position of the shoal was randomized so that in half of the sweeps the virtual shoal was traversed in the bottom half of the tank. In the remaining videos, the virtual shoal was maintained in the bottom or top half of the tank for all the sweeps. When at the boundary of the stimulus side, the shoal image was mirrored so that the orientations of the fish images were always facing the direction of travel. Specifically, the images swam out of the focal region in the screen to enter again by swimming in the opposite direction.

Five minutes long videos were used as “stimuli” in the choice test and were shown simultaneously to a single focal fish for each trial. The same videos were used across the ten trials of each experimental condition. The background of the screen was blue during both the acclimatization and experimental periods.

### Procedure

Experiments were performed in an isolated facility at the Department of Mechanical and Aerospace Engineering at NYU-Poly under controlled conditions.

Ten trials were executed for each experimental condition from April to May 2012. Focal fish were tested from 2 pm to 7 pm. Each fish was tested once. In each experimental condition, the position of the two computer-animated shoals was equally alternated between left and right screen to limit potential confounds. Mosquitofish were manually captured by a net and placed into the test tank. Each trial lasted 15 minutes and consisted of an acclimatization and an observation period. The acclimatization period was ten minutes [33], during which time each focal fish was allowed to swim freely in the test tank while the two screens displayed a blue background. Subsequently, a five minute experimental session was video-recorded to score fish behavior in the presence of the stimuli.

Six experimental conditions were performed in this study to explore the effect of computer-animated images of shoals of mosquitofish altered in color, aspect ratio, and swimming depth on live fish behavior. As sight is the dominant sense in mosquitofish [2],[28], the selection of the experimental conditions was based on [48] where comparable alterations in the appearance of visual stimuli showed increased or reduced preference of zebrafish (whose dominant sensing modality is also vision [28],[57]). In each experimental condition, individual focal fish were shown two different computer-animated shoals on the screens, except for the control condition in which two identical animated shoals were provided. Fish were confronted with the following five pairs of juxtaposed stimuli in which the vertical position of the virtual shoals was randomized in the videos: two unaltered images (Con); unaltered and red colored images (Red); unaltered and yellow colored images (Yel); unaltered and longitudinally elongated images (Elo); and unaltered and longitudinally compressed images (Com) (see Figure 2). In condition Dep, fish were confronted with videos of unaltered images in which the vertical position was kept in the bottom half of the tank on a side and in the top half of the tank of the other.

### Data Acquisition

Fish position was collected through a vision system comprising a computer (Dell, Vostro 220 s, 3 GB of RAM, 2*∶*5 GHz Pentium dual core e5200 processor, Ubuntu 11*:*04 32-bit) and a webcam mounted above the experimental apparatus (Logitech, Webcam Pro 9000). Data acquisition followed the procedure in [41] in which the *x* and *y* positions of the fish were measured relative to the origin *o* of the *xy*-coordinate system located at the center of the experimental tank with axes along the tank walls. A dual-camera setup could have been alternatively used to track three-dimensional positions and bending motions [58]. However, this implementation is limited by considerable computational costs which are not warranted by the selected experimental protocol, which only uses the linear distance between the focal animal and the stimuli [59],[60],[61],[62]. Furthermore, mosquitofish are freshwater fish species that live principally in proximity of the water surface [15],[63] and thus are not expected to considerably vary their swimming depth.

Here, videos were analyzed offline using a custom tracking algorithm to extract fish position data described in [41]. The test tank was virtually divided along its longitudinal axis into three distinct regions following the proportions observed in [52], that is, two stimulus areas 6.5 cm wide proximal to the screens and a third region in the center of the test tank that was 37 cm wide. The time spent by fish in each of these regions was calculated over the five minute experimental session for each trial. Further insight into the interaction between fish and animated images was garnered by analyzing the behavior of mosquitofish using a dedicated software (The Observer 2.0, Noldus, Wageningen, The Netherlands). Based on the literature on the behavior of this species [15],[18],[19],[25],[54],[64], as well as preliminary observations, we scored the following behavioral patterns: “swimming” (the fish moved while not in contact to the stimulus walls), “freezing” (the fish remained completely motionless), and “thrashing” (the fish was moving back and forth against the stimulus walls while its head was physically in contact with the glass).

### Statistical Analysis

For each experimental condition, the statistical significance of fish preference was ascertained using a chi-square test, where the time spent in the two stimulus regions of the test tank were juxtaposed and the expected distribution was taken as uniform (intra-condition preference). Fish preference was the percentage of the time spent in the stimulus area of the altered image out of the time where the fish is present in either stimulus regions (in condition Con, the left stimulus region was taken as “altered” and in condition Dep, the stimulus region for virtual shoals in the tank bottom half was taken “altered”). The variation of observed mean percent preference from the event of no preference was measured for each experimental condition as in [65]. Since this test juxtaposed the two stimuli, one degree of freedom was used.

A one-way analysis of variance (ANOVA) was used for assessing the variation of the total time spent by fish in each of the three regions of the test tank during each five minute experimental trial (inter-condition preference). In this analysis, condition was considered as the independent factor while the time spent by fish in both the stimulus regions and in the center were the dependent variables. The control condition Con was not included in the analysis on fish preference.

A one-way ANOVA was used to investigate both the frequency and duration of the three considered behavioral patterns among the experimental conditions. In other words, frequency and duration of fish behaviors were evaluated for swimming, freezing, and thrashing in each experimental condition. Data analysis was carried out using Statview 5.0. The significance level was set at p≤0.05 for all analyses. Fisher’s protected least significant difference (PLSD) post-hoc tests were used where a significant main effect of the condition variable was observed.

## Results

**Figure 3:**
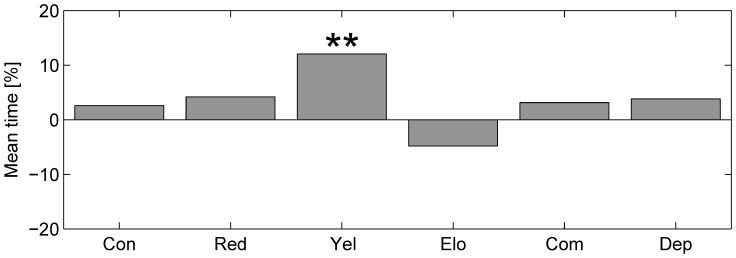
Fish preference for the altered animated shoal. Histograms of fish preference in percentage (∗∗p<0.01). A negative value indicates a preference for the unaltered images.

**Figure 4:**
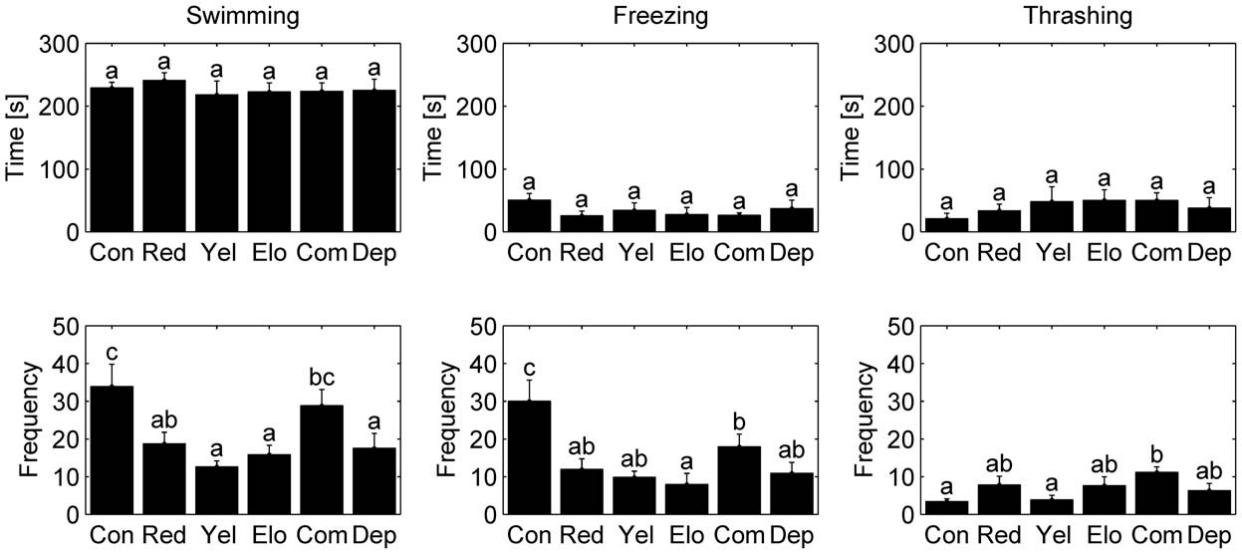
Frequency and duration of fish behaviors. Histograms of time spent (top) and mean number of events (bottom) by fish swimming, freezing, and thrashing, respectively, in the experimental conditions. Error bars refer to the standard error. Means not sharing a common superscript are significantly different (Fisher's PLSD, p<0.05).

We found that mosquitofish preference and behavior were influenced by varying the features of computer-animated images of conspecifics in a canonical dichotomous choice test. Results on preference and behavior are presented in Figure 3 and in Figure 4, respectively. Specifically, we observed that mosquitofish displayed a robust preference for animated images of conspecifics with enhanced yellow pigmentation, and that preference was not influenced by any other alteration. Moreover, the frequencies of the three behaviors were influenced by altering any of the features of the computer-animated images, yet the total time spent exhibiting each behavior was generally unaffected.

### Altered Images: Intra- and Inter-condition Preferences

When considering intra-condition preference, mosquitofish significantly preferred to spend time in the vicinity of the animated images of their conspecifics as their pigmentation is changed to yellow (*χ* ^2^ [1] = 5.818, p≤0.01). Preference was not found to be statistically significant in any other condition (see Figure 3).

When considering inter-condition preference, a significant difference was not observed either in the time spent in the stimulus regions or the central area (data not shown). Investigating into differences between conditions, post-hoc comparisons revealed that the time spent in the altered stimulus region was never significantly different. Condition Red presented the maximum time spent in the center region, that was found to be significantly higher than in conditions Elo and Com (60.0 s and 59.8 s, respectively). Furthermore, condition Elo showed the highest amount of time spent in the unaltered stimulus region and post-hoc comparisons revealed a significant difference compared to conditions Red and Yel (48.6 s and 53.9 s, respectively). Notably, condition Con, in which similar animated stimuli were experimentally compared, was not considered here.

### Behavioral Analysis

Duration and frequency of behaviors were evaluated for each condition (see Figure 4). An effect of the condition was not found in the total time spent swimming. The highest amount of time spent swimming was observed in condition Red (241.0 s) and significant differences between experimental conditions were not found through post-hoc comparisons. Similarly, no condition effect was observed either for the time spent freezing and thrashing with conditions Con and Com showing the highest amount of time (50.6 s and 49.9 s, respectively) spent in these behaviors. Significant differences between experimental conditions were not found through post-hoc comparisons for both freezing and thrashing.

On the other hand, a significant condition effect was found for the frequencies of behaviors. Specifically, a significant condition-effect was found for swimming (F_5,54_ = 4.735, p≤0.01) with the highest number of events observed in condition Con (33.9). Post-hoc comparisons revealed that the swimming frequency in condition Con was significantly higher than conditions Red, Yel, Elo, and Dep and that the swimming frequency in condition Com was significantly higher than conditions Yel, Elo, and Dep. The frequency of freezing was also found to be affected by the condition (F_5,54_ = 5.743, p≤0.01). Specifically, condition Con showed the highest freezing frequency (30.0) and post hoc-comparison revealed that it was significantly different than all the other conditions. Moreover, the number of freezing events in condition Com was also found to be significantly higher than condition Elo. Finally, thrashing behavior was also affected by the condition (F_5,54_ = 2.593, p≤0.05) with condition Com displaying the highest frequency (11.1) and post-hoc comparisons indicating a significant difference with respect to conditions Con and Yel.

## Discussion

The results obtained here demonstrate for the first time that single individuals of the invasive freshwater fish species *Gambusia affinis* [15],[16] respond differently to computer-animated shoals of their conspecifics depending on their color, aspect ratio, and swimming depth in the water column. These findings are consistent with results on the social behavior of comparable visual fish species, such as sticklebacks [42],[66] and zebrafish [41],[48],[49],[50],[52],[53], studied using computer-animated images. Specifically, phenotypic varieties of zebrafish were found to react differently to animated images of their conspecifics depending on similarities of their stripe patterns [48],[52] and color pigment [48]. Similar results on zebrafish shoaling preference were obtained in other studies using canonical preference tests with live stimuli [59] and bioinspired robots [65],[67].

As depicted in Figure 3, mosquitofish are strongly attracted to animated images of their conspecifics when such images are artificially altered to exhibit an increased yellow pigmentation. The importance of pigmentation in choice behavior of fish is widely documented in the literature [68] and attraction towards yellow-colored computer-animated images of conspecifics was also found in zebrafish in [48]. Therein, such attraction was attributed to good health conditions and reproductive maturity signaled by yellow pigmentation of zebrafish. Large amounts of yellow pigmentations in male mosquitofish are generally related to higher social ranks in mosquitofish populations [32]. Specifically, dominant mosquitofish males can be visually identified based on the abundance of yellow pigment on their dorsal fin and along the dorso-lateral region of the body [32]. Social hierarchy in males is also a determinant of the attraction of mosquitofish females for them [26], which also rests upon melanism [64]. Mosquitofish females tend to avoid sexual harassment of subordinate males (by reducing their inter-individual distance) [25],[26],[54],[69], while they are attracted towards dominant males. These social behaviors can be interpreted as the result of the balance between costs (higher competition for food and parasite transmission) and benefits (diluted sexual harassment) of shoaling in female mosquitofish [26]. In this direction, we interpret the observed preference of female mosquitofish for the yellow colored images as the natural consequence of their attraction towards dominant males. On the contrary, we speculate that the abundance of yellow pigmentation could result in an aversive response of subordinate males, avoiding the aggressive dominant males [26],[32].

Mosquitofish are never significantly influenced negatively in their preference by altered images (see Figure 3) even those evoking typical small freshwater fish predators, such as the elongated conspecific [48], or those addressing species that are unlikely to be present in freshwater environments, such as the red colored conspecific [48]. This finding seems to corroborate results in [19], where it was observed that predation risk does not influence either the foraging success or the behavior of *Gambusia affinis* in the presence of a predator fish species. In fact, differently from other fish of comparable size, mosquitofish reflect both the physiological and the morphological characteristics of a generalist predatory fish species [13],[15],[70]. The high competitive and aggressive behavior of mosquitofish towards other species of fish [18],[21],[22] and amphibians [13],[17] of similar size play an important role in their ecological success [33].

A large variation of behavioral activity is found for mosquitofish as the computer-animated images are changed. The relatively small frequencies of the three behaviors for condition condition Yel, as compared with the control condition condition Con, indicate that female mosquitofish tend to steadily interact with the yellow colored images without displaying sudden changes in their locomotory pattern observed in other conditions (see Figure 4). Similar results are found in [48] for zebrafish.

The results of this study may offer further insight into the social behavior of an ecologically problematic animal species [2],[13],[16], that is receiving an increasing interest from the scientific community for its negative impact on economy and biodiversity [4],[5]. Exploring the determinants of social response in mosquitofish at a species-specific level may inform the design of control strategies and open new avenues in conservation and protection of endangered animal species. For example, these findings could be integrated in the design of self-propelled bioinspired robotic-fish [65],[67] for deployment in wild ecological communities to modulate mosquitofish behavior and protect relevant nesting sites and nursery areas of native species.
